# Knowledge and Attitude Toward Antibiotic Prescription Among Dental Students and Interns at Multiple Universities in Saudi Arabia

**DOI:** 10.7759/cureus.51777

**Published:** 2024-01-07

**Authors:** Arwa Mubarak, Malak M Alwafi, Rahaf M Alharbi, Sarah A Alserhani, Raghad F Khushaim, Ghusun Z Almadani, Ibrahim M Nourwali, Muath S Alassaf

**Affiliations:** 1 Dentistry, Taibah University, Madinah, SAU; 2 Pediatric Dentistry and Orthodontics, Taibah University, Madinah, SAU; 3 Department of Dental Education, Taibah University, Madinah, SAU; 4 Department of Oral and Maxillofacial Surgery, Taibah University, Madinah, SAU; 5 Orthodontics and Dentofacial Orthopedics, Taibah University, Madinah, SAU

**Keywords:** prophylactic antibiotics, attitude, knowledge, periodontal disease, antibiotic policies and guidelines

## Abstract

Background: Dental students in Saudi Arabia are authorized to write prescriptions for antibiotics during practical training. Adverse side effects and resistance could result from inappropriate prescription. Accordingly, there is a need to evaluate the knowledge of dental students regarding guidelines and applications of antibiotic prescription.

Objectives: To assess the knowledge and attitude toward guidelines and applications of antibiotic prescription among dental students and interns at multiple universities in Saudi Arabia.

Methods: A cross-sectional study was conducted among dental students in their final clinical years (4th to 6th year) and dental interns. The study data were collected using a valid and reliable structured questionnaire comprising three domains: 1) demographic characteristics, 2) knowledge, and 3) attitude toward antibiotic prescription for dental and systemic conditions. The data were analyzed and presented as frequency percentages, and the chi-square test was used to compare the knowledge and attitude items between the dental students and interns. The statistical significance level was set at p ≤ 0.05.

Results: A total of 248 participants (women: 55.6%, men: 44.4%) were included in the study. Approximately 21.8% were 4th year students; 17.7%, 5th year students; 12.9%, 6th year students; and 47.6%, interns. For most items, the knowledge level was relatively high, and the attitude was generally positive among the participants. Approximately, 87.1% had good knowledge about current guidelines for antibiotic prophylaxis, 83.9% about antibiotic prescription, and 95.2% about antibiotic resistance. The interns showed significantly higher knowledge levels and favorable attitude, particularly for guidelines and applications of antibiotic prescription and correct use of antibiotics for oral cases, than did the students. Amoxicillin was the most frequently prescribed antibiotic among the participants.

Conclusion: The interns and 6th-year students demonstrated a relatively high knowledge level and positive attitude toward appropriate antibiotic prescriptions. However, deficiencies were observed among the students in their early clinical years, particularly for systemic conditions. These findings highlight the importance of implementing educational campaigns and providing guidelines to promote the appropriate use of antibiotics among dental students in their final clinical years.

## Introduction

Since Alexander Fleming introduced penicillin in 1928, antibiotics have become widely used in medicine [1]. Antibiotics are primarily used to treat bacterial infections by killing or inhibiting the growth of bacteria [2]. However, one of the challenges faced by clinicians after the introduction of antibiotics is the emergence of bacterial resistance to these drugs [3]. In the field of dentistry, antibiotics are primarily utilized for treating infections associated with dental issues. However, recent research has shown that many endodontic infections can be managed without antibiotics through local interventions such as incision and drainage, root canal treatment, or tooth extraction [2,4].

To promote appropriate antibiotic prescription practices, the World Health Organization, the Faculty of General Dental Practice in the United Kingdom, and the Scottish Dental Clinical Effectiveness Program have issued guidelines. These guidelines emphasize that antibiotics are not recommended for non-spreading infections of the teeth and alveolar bone in healthy individuals [5-7].

Despite the accumulating evidence of antimicrobial resistance, dentists and dental students have been shown to still lack sufficient knowledge in this area [8-11]. In Saudi Arabia, several studies have assessed the knowledge and attitude toward antibiotic prescription among dental students [2,12-15]. These studies have revealed that dentists have an intermediate knowledge level of antibiotic prescription guidelines and often prescribe antibiotics even when they are not necessary and that amoxicillin-clavulanate is the most commonly prescribed antibiotic for endodontic infections [2,12-16]. Additionally, a survey among non-medical female university students in Riyadh showed misconceptions and negative attitudes toward antibiotic use for dental treatment [17].

In Saudi Arabia, dental students are authorized to prescribe antibiotics under the supervision of their instructors during their clinical training years [18,19]. Attitude toward prescribing medications and experiences with specific antibiotics are developed during this training period and can influence clinical practice. Therefore, it is crucial to instill concepts of antibiotic resistance and appropriate prescription indications early on. The American Association of Endodontists has published guidelines on the use of antibiotics for the treatment of endodontic infections, and these guidelines are commonly followed in Saudi Arabia [20,21].

While several studies have assessed antibiotic-related knowledge and prescription patterns among practicing dentists, few have focused on dental students in different academic years and interns. Accordingly, this study aimed to assess the knowledge and attitude toward guidelines and applications of antibiotic prescription among dental students in different academic years and dental interns at multiple universities in Saudi Arabia.

## Materials and methods

A cross-sectional study was conducted among dental students in their final clinical years (early: 4th and 5th years, senior: 6th year) and dental interns. The sample size was calculated using the Epi Info program, and based on the prevalence of familiarity with the concept of antibiotic resistance (71%) among dental students in a previous similar Saudi study, an alpha error of 5%, and a confidence limit of 95% [16]. Accordingly, the sample size was calculated to be 300.

A validated questionnaire adapted from the study conducted by AboAlSamh et al. was used [16]. The questionnaire collected information on demographic characteristics (sex, college, grade of study/intern), knowledge, and attitude toward antibiotic prophylaxis, antibiotic resistance, antibiotic prescription guidelines, clinical conditions for which antibiotics are indicated, commonly prescribed antibiotics, and antibiotic regimen durations. The knowledge was assessed by 26 questions with two answers for each question (yes, no). Each knowledge item was scored as follows: correct answer = 1, and incorrect answer = 0. The attitude was assessed by six questions including two multiple-choice and four closed-ended questions. As English is the primary language of instruction in Saudi Arabia, translation of the questionnaire was not required. The questionnaire was converted into an electronic form using Google Forms, a free web-based survey generator, to facilitate data collection. The electronic version maintained the same format as the original paper questionnaire, preserving the options and answering fields. To prevent duplicate responses, Google Forms’ built-in limit to one response and response validation were used.

A link to the questionnaire was generated and distributed through various social media platforms, including Twitter, Telegram, Instagram, and WhatsApp. The survey was shared with dental students and interns from the following dentistry colleges at universities in Saudi Arabia: Taibah, Um Alqura, King Abdulaziz, King Saud, King Khalid, King Faisal, Hail, Qassim, Dammam, Imam Abdulrahman bin Faisal, Jazan, Dar Al-Uloom, Riyadh Elm, Majmaah, Princess Nora bint Abdulrahman, Vison, and Ibn Sina National College. These universities were chosen from a selection of universities in the kingdom that have a faculty of medicine as part of their academic discipline.

The study procedures were reviewed and approved by the Research Ethics Committee of Taibah University, College of Dentistry (Approval number: TUCDREC/051022/AMMubarak). Participation in this study was voluntary. The privacy and confidentiality of the participants were ensured by collecting and manipulating the data anonymously. The study's aim and scope were carefully explained to the participants.

The collected data was inputted into an Excel file and subsequently transferred for analysis using the Statistical Package for the Social Sciences (version 22.0; SPSS Inc., Chicago, IL). The personal data of the participants was tabulated and presented as numbers and frequency percentages. The participants’ knowledge levels and attitudes toward guidelines and applications of antibiotic prescription were estimated and compared according to their academic level using the chi-square and Fischer’s exact tests as appropriate. P-values of ≤0.05 were considered to indicate statistical significance.

## Results

A total of 248 participants (women: 55.6%, men: 44.4%) were included in the study. Approximately 21.8% were 4th-year students; 17.7% were 5th-year students; 12.9% were 6th-year students; and 47.6% were interns (Table 1). The overall response rate for participation in this study was 89%.

**Table 1:** Demographic data (N = 248)

	Variable	N	%
Sex	Female	138	55.6
	Male	110	44.4
Academic level	4th-year student	54	21.8
	5th-year student	44	17.7
	6th-year student	32	12.9
	Intern	118	47.6

Table 2 shows the responses of the participants about their knowledge of antibiotics. Approximately 87.1% of the participants reported being aware of and following antibiotic prevention instructions. The majority (95.2%) indicated their knowledge of antibiotic resistance. Approximately 61.3% were aware that antibiotic resistance is developed by microorganisms in response to exposure to antibiotics; 14.9% said that in cases of antibiotic resistance, the body does not respond to antibiotics; 10.5% said that an inadequate dosage of antibiotics can cause resistance; and 9.7% said that infections can persist despite high doses of antibiotics.

**Table 2:** Knowledge about antibiotic resistance (N = 248)

Knowledge	Answer	n	%
Are you aware of the currently used Saudi guidelines for antibiotic prophylaxis, and do you follow the same?	Yes	216	87.1
	No	32	12.9
Are you aware of antibiotic resistance?	Yes	236	95.2
	No	12	4.8
What is your understanding of antibiotic resistance?	Infections can persist despite high doses of antibiotics.	24	9.7
	An inadequate dosage of antibiotics can cause resistance.	26	10.5
	Resistance is developed by microorganisms in response to exposure to antibiotics.	152	61.3
	The body does not respond to antibiotics.	37	14.9
Are you aware that self-medication with antibiotics for relief of dental pain can cause antibiotic resistance?	Yes	214	86.3
	No	34	13.7
Are you aware of the guidelines for antibiotic prescription?	Yes	208	83.9
	No	40	16.1
What is the duration of the antibiotic course?	3 days	30	12.1
	5 days	218	87.9

The knowledge level of the participants about the risks of self-medication with antibiotics was high, with 86.3% of them agreeing that self-medication for dental pain relief can cause antibiotic resistance. Their knowledge level of antibiotic prescription instructions was also high (83.9%). Approximately 87.9% of the participants answered that the course of antibiotics is 5 days, while 12.1% answered 3 days.

Table 3 shows the participants’ responses about prescribing antibiotics in routine cases. Approximately 92.7% and 82.7% of the participants reported not prescribing antibiotics for reversible pulpitis and irreversible pulpitis, respectively. Conversely, 69% agreed with prescribing antibiotics for the extraoral draining sinus tract and only 50% for intraoral draining sinus tract. Of the participants, 65.7% indicated not prescribing antibiotics for localized intraoral swelling, while 93.1% reported prescribing such for acute facial swelling. Approximately 71% agreed with prescribing antibiotics for dental trauma, 48.8% for pericoronitis, 66.9% for extraction via the open method, and 46.4% for periapical abscess. In contrast, 79.4% disagreed with prescribing such for pediatric periodontal diseases, 94% for simple extraction, 76.2% for apical periodontitis, and 66.1% for dry sockets.

**Table 3:** Responses to items related to antibiotic prescription for medical conditions (N= 248)

Case requiring antibiotic prescription		n	%
Reversible pulpitis	Yes	18	7.3
	No	230	92.7
Irreversible pulpitis	Yes	43	17.3
	No	205	82.7
Extraoral draining sinus tract	Yes	171	69.0
	No	77	31.0
Intraoral draining sinus tract	Yes	124	50.0
	No	124	50.0
Localized intraoral swelling	Yes	85	34.3
	No	163	65.7
Acute facial swelling	Yes	231	93.1
	No	17	6.9
Dental trauma	Yes	72	29.0
	No	176	71.0
Pediatric periodontal diseases	Yes	51	20.6
	No	197	79.4
Pericoronitis	Yes	121	48.8
	No	127	51.2
Simple extraction	Yes	15	6.0
	No	233	94.0
Extraction via the open method	Yes	166	66.9
	No	82	33.1
Periapical abscess	Yes	115	46.4
	No	133	53.6
Apical periodontitis	Yes	59	23.8
	No	189	76.2
Dry sockets	Yes	84	33.9
	No	164	66.1

As shown in Table 4, 73% of the participants reported not prescribing antibiotics for viral infections, 72.2% for juvenile diabetes, 70.2% for blood dyscrasias, and 75.4% for respiratory disorders.

**Table 4:** Responses to items related to antibiotic prescription for systemic conditions (N = 248)

Case		n	%
Viral infections	Yes	67	27.0
	No	181	73.0
Juvenile diabetes	Yes	69	27.8
	No	179	72.2
Blood dyscrasias	Yes	74	29.8
	No	174	70.2
Respiratory disorders	Yes	61	24.6
	No	187	75.4

**Figure 1:**
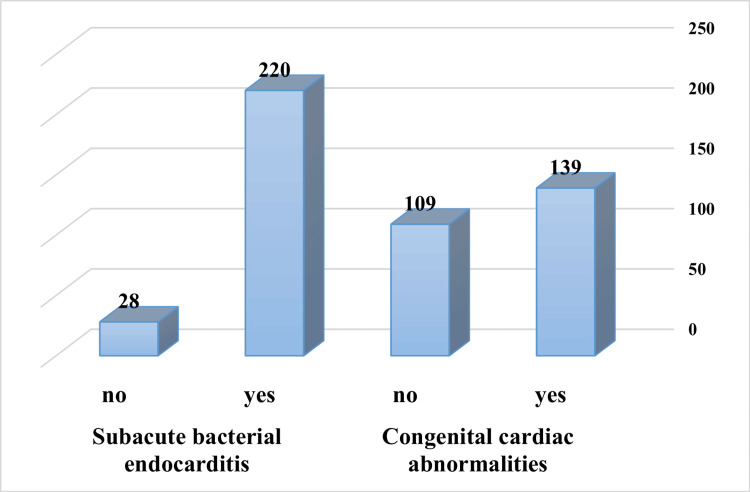
Responses to items related to antibiotic prescription for cardiovascular diseases

The participants’ responses about prescribing prophylactic antibiotics for cardiovascular diseases are presented in Figure 1. About half of the respondents (56%) reported prescribing antibiotics for congenital cardiac abnormalities and 88.7% for subacute bacterial endocarditis.

Table 5 presents the attitude of the participants regarding prescribing antibiotics. The majority of the participants (94.4%) reported that the most commonly prescribed antibiotic was amoxicillin; 2.8%, clindamycin; 1.6%, cephalexin; and 1.2%, ofloxacin with ornidazole.

**Table 5:** Attitude toward antibiotic prescription (N = 248)

Attitude		n	%
Which antibiotic do you prescribe the most?	Amoxicillin	234	94.4
	Ofloxacin with ornidazole	3	1.2
	Cephalexin	4	1.6
	Clindamycin	7	2.8
How do you decide which antibiotic to use?	Based on symptoms	61	24.6
	Based on guidelines	181	73
	Based on the cost of the drug	6	2.4
Do you ask your patient whether he/she has taken a course of antibiotics in the past 1 week before prescribing antibiotics?	Yes	193	77.8
	No	55	22.2
Do you advise your patient to adhere to the dosage regimen and inform them of the consequences of not doing so?	Yes	220	88.7
	No	28	11.3

Regarding attitude toward decisions for prescribing appropriate antibiotics, most of the participants (73%) indicated prescribing them based on guidelines, 24.6% based on symptoms, and only 2.4% based on the cost of the drug. When asked whether the patients taking antibiotics in the past week before prescribing new antibiotics, 77.8% answered yes, while 22.2% answered no. When asked whether they advise patients about committing to the antibiotic dosage regimen and the consequences of doing otherwise, 88.7% answered yes, while 11.3% answered no.

**Figure 2:**
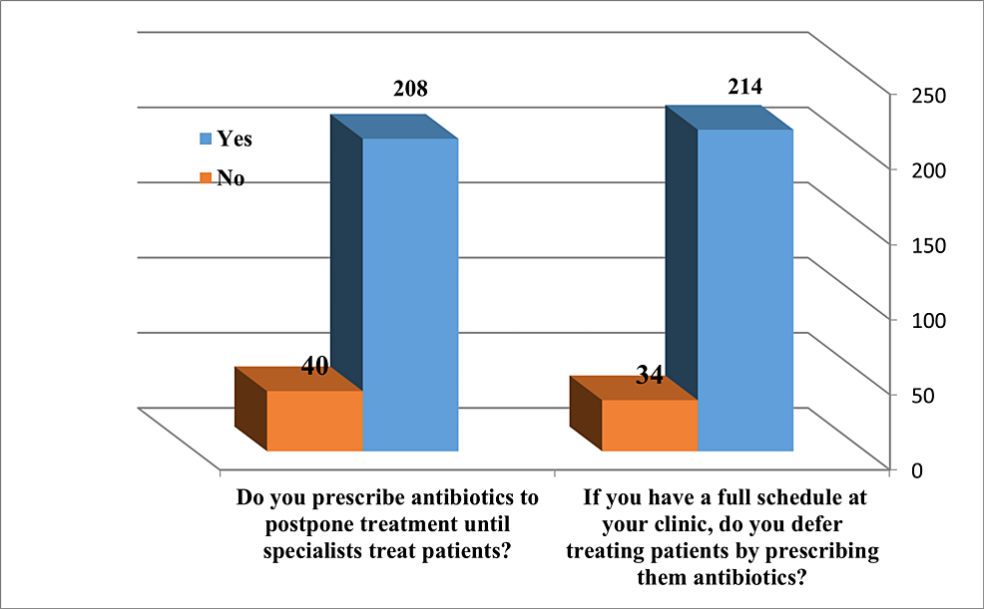
Responses and attitude toward antibiotic prescription in some cases

The participants’ responses and attitudes about prescribing antibiotics in some cases are presented in Figure 2. Approximately 86.3% answered that they do not delay the treatment of patients by using antibiotics in case of a full schedule, while 13.7% answered that they prescribe antibiotics to postpone treatment until specialists treat patients. Further, 83.9% indicated prescribing antibiotics to save a patient’s life.

Table 6 presents the comparison of the knowledge among the participants according to their academic level. There were significant differences noted in the knowledge level among the participants. The interns showed the highest level of knowledge, followed by the 6th-year students, while the 4th-year students showed the lowest level of knowledge for most items.

**Table 6:** Comparison of the knowledge among the participants according to their academic level *P-value equal or less than 0.05

Knowledge		Academic level				p-value
		4th year students (n = 55)	5th year students (n =44)	6th year students (n = 32)	Interns (n = 118)	
Are you aware of the current guidelines for antibiotic prophylaxis, and do you follow the same?	Yes	39	37	29	111	0.001^*^
	No	15	7	3	7	
Are you aware of the guidelines for antibiotic prescription?	Yes	38	37	26	107	0.009^*^
	No	16	7	6	11	
What is the duration of the antibiotic course?	3 days	18	4	0	8	0.000^*^
	5 days	36	40	32	110	
Reversible pulpitis	Yes	13	0	1	4	0.000^*^
	No	41	44	31	114	
Irreversible pulpitis	Yes	26	2	2	13	0.000^*^
	No	28	42	30	105	
Extraoral draining sinus tract	Yes	43	25	15	41	0.000^*^
	No	11	19	17	77	
Intraoral draining sinus tract	Yes	43	25	15	41	0.000^*^
	No	11	19	17	77	
Simple extraction	Yes	8	2	2	3	0.018^*^
	No	46	42	30	115	
Viral infections	Yes	25	6	10	26	0.001^*^
	No	29	38	22	92	
Respiratory disorders	Yes	20	13	6	22	0.047^*^
	No	34	31	26	96	
Subacute bacterial endocarditis	Yes	46	34	29	111	0.019^*^
	No	8	10	3	7	

As shown in Table 7, there were significant differences in nearly all attitude items among the participants according to their academic level. The interns and 6th-year students had the most positive attitude, particularly toward prescriptions based on guidelines and postponement of treatment until specialists treat patients. Almost all participants reported amoxicillin as the most commonly prescribed antibiotic.

**Table 7:** Comparison of the attitude among the participants according to their academic level * P-value equal or less than 0.05

Attitude		Academic level				p-value
		4th-year students (n = 55)	5th-year students (n = 44)	6th-year students (n = 32)	Interns (n = 118)	
Which antibiotic do you prescribe the most?	Amoxicillin	45	42	32	115	0.011^*^
	Ofloxacin with ornidazole	3	0	0	0	
	Cephalexin	2	0	0	2	
	Clindamycin	4	2	0	1	
How do you decide which antibiotic to use?	Based on symptoms	26	10	8	17	0.001^*^
	Based on guidelines	27	33	24	97	
	Based on the cost of the drug	1	1	0	4	
If you have a full schedule at your clinic, do you defer treating patients by prescribing them antibiotics?	Yes	13	4	4	9	0.020^*^
	No	41	40	28	109	
Do you prescribe antibiotics to postpone treatment until specialists treat patients?	Yes	19	17	9	20	0.011^*^
	No	35	27	23	98	

## Discussion

This study revealed a high percentage of dental students and interns demonstrated knowledge about current guidelines for antibiotic prophylaxis and prescription. Previous similar studies in Saudi Arabia, however, have indicated a lack of adequate knowledge [13,15]. The majority of participants in the present study showed a good understanding of antibiotic resistance. This finding was also higher than that reported in another Saudi study [16]. Most oral conditions encountered in dental clinics are inflammatory in nature and typically require operative interventions rather than antibiotics. Despite guidelines suggesting that antibiotics are unnecessary for these conditions, they are often prescribed, although their actual benefit remains uncertain [20]. For reversible pulpitis, irreversible pulpitis, and dry sockets, the majority of participants reported not prescribing antibiotics, and this aligns with the recommended approach. However, previous research has shown that antibiotics are frequently prescribed for these conditions [22].

Another important finding in this study is that about one-fourth of the participants would recommend antibiotics for viral infections, despite the fact that symptomatic relief is the preferred treatment for viral infections [23]. The guidelines from the American Heart Association and American College of Cardiology recommend antibiotic prophylaxis for certain patients undergoing invasive dental procedures, including those with specific cardiac conditions [21]. The majority of participants (88.7%) in this study would prescribe antibiotics as prophylaxis for patients with subacute bacterial endocarditis, which is higher than the percentage reported in a similar Saudi study [16].

In dental practice, penicillin is the most commonly prescribed antibiotic [24,25]. Consistent with other studies, the current study found that amoxicillin was the preferred antibiotic choice prescribed by the participants [9,13,15,16,24-26]. The recommended duration of antibiotic treatment in therapeutic guidelines is often based on expert opinions [27]. The study findings revealed that the duration of antibiotic treatment prescribed by participants was five days. However, three days were advised for the treatment of acute dento-alveolar infections [28]. Previous studies found that antibiotics are typically prescribed for an average of 6.9 days and 7.6 days, respectively [29,30].

The study's strengths include a high response rate, a valid questionnaire, and a diverse sample from different universities in Saudi Arabia. The study not only assessed awareness levels but also compared knowledge and attitude based on the participants' academic level.

Limitation of the study

The limitation of this study is that it primarily relied on quantitative data, which limited the depth of understanding of participants' attitudes. Additionally, there may have been self-selection bias, as the dental students and interns who chose to participate might have had a better understanding of the concepts compared to those who refused to be contacted. However, the low refusal rate in this study suggests that self-selection bias did not significantly impact the study findings.

## Conclusions

In conclusion, the findings of this study indicate that senior (6th-year) dental students and interns generally have good knowledge levels and positive attitudes toward guidelines and applications of antibiotic prescriptions for oral cases. Conversely, early (4th-year) final clinical year students have a moderate-to-low level of knowledge in this area. Dental students and interns appear to inappropriately prescribe antibiotics to manage systemic conditions, even when they are not indicated. These findings highlight the need for urgent educational campaigns and the provision of guidelines to promote the rational use of antibiotics among early-final clinical-year dental students. It is important to disseminate the findings of this study to improve the quality of dental programs in the early final clinical years. Future studies should focus on examining the reasons behind the varying attitudes of dental students and interns, and it would be beneficial to adopt a large-scale national longitudinal design for this purpose.

## Appendices

Part I: Demographic information

1.    Gender

o   Male

o   Female

2.    What university/college are you enrolled in?

o   Taibah University

o   King Abdulaziz University (KSU)

o   Ibn Sina National Collage for Medical Studies

o   King Saud bin Abdulaziz University for Health Sciences (KSAU-HS)

o   Riyadh Elm University (REU)

o   King Khalid University (KKU)

o   Princess Nora bint Abdulrahman University (PNU)

3.    What year are you enrolled in at your college?

o   4th year

o   5th year

o   6th year

o   Intern

Part II: Knowledge

1.    Are you aware of the current guidelines for “antibiotic prophylaxis” and do you follow the same?

o   Yes

o   No

2.    Are you aware of the term “antibiotic resistance”?

o   Yes

o   No

3.    If yes, what do you understand by “antibiotic resistance”?

o   Infection is not under control even after taking high doses of antibiotics

o   Inadequate dosage of antibiotics cause resistance

o   Resistance acquired by microorganism to antibiotics

o   Body does not respond to antibiotics

4.    Self-medication with antibiotics by patients to get relief from dental pain may be responsible for antibiotic resistance.

o   Yes

o   No

5.    Are you aware of the guidelines for “antibiotic prescription”?

o   Yes

o   No

6.    What is the duration of the antibiotic course?

o   3 days

o   5 days

•       Would you routinely prescribe antibiotics in the following situations?

7.    Reversible pulpitis:

o   Yes

o   No

8.    Irreversible pulpitis:

o   Yes

o   No

9.    Extraoral draining sinus tract:

o   Yes

o   No

10. Extraoral draining sinus tract:

o   Yes

o   No

11. Localized intraoral swelling:

o   Yes

o   No

12. Acute facial swelling:

o   Yes

o   No

13. Dental trauma:

o   Yes

o   No

14. Pediatric periodontal diseases:

o   Yes

o   No

15. Pericoronitis:

o   Yes

o   No

16. Simple extraction:

o   Yes

o   No

17. Extraction by open method:

o   Yes

o   No

18. Periapical abscess:

o   Yes

o   No

19. Apical periodontitis:

o   Yes

o   No

20. Dry socket:

o   Yes

o   No

•       Would you prescribe antibiotics for the following systemic conditions?

21. Viral infections:

o   Yes

o   No

22. Juvenile diabetes:

o   Yes

o   No

23. Blood dyscrasias:

o   Yes

o   No

24. Respiratory disorders:

o   Yes

o   No

•       Would you prescribe prophylactic antibiotics in cases of cardiovascular diseases such as?

25. Congenital cardiac abnormalities:

o   Yes

o   No

26. Subacute bacterial endocarditis:

o   Yes

o   No

Part III: Attitude

1.    Which is the most commonly prescribed antibiotic by you?

o   Amoxicillin

o   Ofloxacin with ornidazole

o   Cephalexin

o   Clindamycin

2.    How do you most of the time decide which antibiotic to use?

o   Based on symptoms

o   Based on guidelines

o   Based on the cost of the drug

3.    Do you inquire from your patient about whether he/she has taken a course of antibiotics in the past 1 week before prescribing antibiotics?

o   Yes

o   No

4.    Do you advise your patient to adhere to the dosage regimen and inform the consequences of not doing so

o   Yes

o   No

•       Do you prescribe antibiotics in these situations?

5.    If you have a full schedule at your clinic, do you defer treating patients by putting them on antibiotics?

o   Yes

o   No

6.    Do you prescribe antibiotics to sustain the patient until the specialist treats the patient?

o   Yes

o   No
